# Periodic Leg Movements during Sleep in Japanese Community-dwelling Adults Based on the Assessments of Their Bed Partners

**DOI:** 10.2188/jea.13.259

**Published:** 2007-11-30

**Authors:** Yuriko Doi, Yuichi Inoue, Masumi Minowa, Makoto Uchiyama, Masako Okawa

**Affiliations:** 1 Department of Epidemiology, National Institute of Public Health.; 2 Japan Somnology Center Neuropsychiatry Institute.; 3 Department of Psychophysiology, National Institute of Mental Health.; 4 Department of Psychiatry, Shiga University of Medical Science.

**Keywords:** epidemiology, periodic limb movements, periodic leg movements during sleep, sleep

## Abstract

BACKGROUND: There is little known about epidemiologic evidence on periodic leg movements during sleep (PLMS) for the Japanese. The present study was a cross-sectional epidemiologic study to estimate the prevalence of PLMS and examine the associated factors of PLMS in Japanese community-dwelling adults.

METHODS: The subjects were 884 with bed partners or bedroom mates of 1,889 Japanese adults aged 20 years and over randomly selected from the general population. The case ascertainment of PLMS was based on the assessments of their bed partners or bedroom mates using the Pittsburgh Sleep Quality Index. Multiple logistic regression analyses were used for investigating the associated factors.

RESULTS: The age-adjusted prevalences (95% confidence interval) were 5.8% (4.7-6.8%) and 1.3% (0.8-1.9%) for 1 to 2-times, and 3-times or greater of PLMS per week during the preceding month, respectively. Those with PLMS were more likely to experience difficulty in initiating sleep, snore during sleep, be depressed, and suffer from peptic ulcer. Sex, age, difficulty in maintaining sleep, excessive daytime sleepiness, medication use to aid sleep, and any psychoactive substances (tobacco, alcohol, and caffeine) were not identified as significant associated factors of PLMS.

CONCLUSIONS: The results suggest that the prevalence of PLMS in Japanese community-dwelling adults is not so high as those reported from Western countries, and that PLMS is correlated with some sleep and health disturbances.

Periodic limb movements during sleep, which was originally described as nocturnal myoclonus by Symonds,^1^ is characterized by relatively stereotyped repetitive movements in limbs occurring during sleep.^2^^-^^6^ Periodic limb movements are best described as rhythmical extensions of the big toe and dorsiflextions of the ankle with occasional flexions of the knee and hip. The movements usually occur in the legs and similar movements can occur in the upper limbs.^6^^,^^7^ The periodic leg movements during sleep (PLMS) are a characteristic feature of periodic limb movement disorder.^8^ The periodic limb movement disorder is a relatively new clinical entity, and the prevalence and predisposing factors still remain to be investigated.

Previous studies on periodic limb movements during sleep reported a prevalence of 11-13% in sleep disorder patients,^9^^,^^10^ 11% (6% nocturnal myoclonus and 5% nocturnal myoclonic activity) in healthy adults,^11^ 10-16% in randomly selected elders and 45% in elderly volunteers willing to have their sleep recorded,^12^ and 2-12% in adult community residents.^13^^,^^14^ They also examined the associations with age, sex, sleep disturbances, daytime sleepiness, napping, sleep apnea or breathing problems, hypertension, mood or affective disorders, psychoactive substances (alcohol, caffeine, tobacco and central nervous system medication), occupation, and low household income,^9^^-^^22^ but the findings obtained across studies were not consistent.

The findings of most previous studies came from those who were clinically referred or willing to be tested. More epidemiologic research on periodic limb movement disorder was needed for the non-referred subjects of a target study population. For one reason, we wanted to know how prevalent it was among the non-referred subjects from the viewpoints of public health and health education, because it was not known well in society and those with periodic limb movement disorder accordingly might not be identified or treated appropriately. We also wanted to make a part of the investigations on its risk factors for further research on the etiology. The purpose of the present study was to estimate the prevalence of PLMS, a characteristic feature of period limb movement disorder, and examine its associated factors in Japanese community-dwelling adults based on the assessments of their bed partners or bedroom mates.

## METHODS

### Subjects and Data Collection

A nationwide epidemiologic study on sleep and health was conducted for 2,800 Japanese adults aged 20+ years randomly selected from the residential registries based on the 1995 Census. A self-administered questionnaire was mailed to each subject in autumn, 1997. Two reminders were sent at intervals of a few weeks to subjects who had not replied within two weeks. The participants for the study were 1,889 respondents who basically agreed with voluntary and anonymous participation in this study (response rate: 67.5%). The method used for this survey was previously reported in detail elsewhere.^23^

The subjects used for analyses were 884 with bed partners or bedroom mates: sex, 441 males and 443 females; age, 59, 178, 174, 155, 138, 113, and 67 for the age groups of 20-29, 30-39, 40-49, 50-59, 60-69, 70-79, and 80+ years, respectively; marital status, 830 married, 50 unmarried, and 4 unascertained; educational status, 249 junior high school graduates, 629 university, college, technical school or high school graduates, and 6 unascertained; employment status, 546 employed, 333 unemployed, and 5 unascertained.

### Questionnaire

A self-administered questionnaire used for the present study consisted of 60 items on (1) sociodemographic information (e.g., sex, age, marital status, educational attainment, and job status), (2) physical health status on present medical illnesses (e.g., hypertension, heart disease, stroke, diabetes mellitus, liver disease, renal disease, asthma, peptic ulcer and others), utilization of medical services or consultations, and activities of everyday life, (3) mental health status using the Japanese version of the 12-item General Health Questionnaire,^24^^-^^26^ (4) sleep habit and sleep problems using the Japanese version of Pittsburgh Sleep Quality Index,^27^^,^^28^ (5) the environmental conditions of noise, light, temperature, moisture, and space in a bedroom, and (6) psychoactive substance use affecting sleep (e.g., tobacco, alcohol, and caffeine-contained beverages).

### Case Ascertainment

PLMS and sleep-related complaints were assessed with the following respective items drawn from the Pittsburgh Sleep Quality Iindex.

1) PLMS: if you have a bed partner or bedroom mate, ask him/her how often you have had legs twitching or jerking while you sleep.
2) Difficulty in initiating sleep; how often have you had trouble sleeping because you cannot get to sleep within 30 minutes?
3) Difficulty in maintaining sleep: how often have you had trouble sleeping because you wake up in the middle of the night or early morning?
4) Excessive daytime sleepiness: how often have you had trouble staying awake while driving, eating meals, or engaging in social activity?

The subjects were asked to choose one response from the following four answers: (1) not at all in one week, (2) less than once a week, (3) once or twice a week, and (4) three or more times a week in the previous month. Those who replied not at all per week in the past month to all of the above items were judged not to suffer from PLMS or sleep-related complaints. The rest were categorized as those with PLMS, the difficulty in initiation sleep, difficulty in maintaining sleep, and the excessive daytime steepness.

The case ascertainment applied for this study was based on symptoms alone. Clinical diagnoses including polysomnographic and electromyographic recordings,^27^ in the steep laboratory could not be incorporated into a large-scale community-based epidemiologic study.

### Statistical Analysis

Statistical analysis was performed for the subjects with bed partners or bedroom mates. The age-adjusted prevalence with a 95% confidence interval (CI) of PLMS and sleep-related complaints (difficulty in initiating sleep, difficulty in maintaining steep, and excessive daytime sleepiness) were estimated, after being weighted according to the proportion of each age group in the total adult population determined by the 1995 Census. The number of those with PLMS was so small that the age-adjusted prevalence of PLMS was calculated for the combined data of males and females. Sex difference in PLMS was investigated using logistic regression models.

To examine the associated factors with PLMS, a multiple logistic regression analysis was used.^29^ A univariate analysis was performed to detect the association of PLMS with each of the variables, and then the variables were analyzed using the forward selection stepwise procedure (P≦0.05 as inclusion and P≧0.10 as exclusion). Strong intercorrelations between variables were checked and excluded from a multiple logistic regression model. The variables entered in the models (the reference groups) were determined based on the previously reported studies;^9^^-^^22^ (1) sociodemographic variables: sex (males), age group (20-29 years old), marital status (unmarried), educational status (higher educational attainment above high school), and job status (employed), (2) sleep-related complaints: difficulty in initiating sleep, difficulty in maintaining sleep, excessive daytime sleepness, and snoring (absent: not at all in the past month), (3) medication use to help sleep (absent: not at all in the past month), (4) physical health variables: present medical illnesses (absent), (5) mental health variables: each item of the 12-item General Health Questionnaire excluding the item on insomnia (absent), and (6) psychoactive substance use affecting sleep: smoking (never), alcohol drinking (never) and caffeine drinking (never).

Subjects with missing values were eliminated from the analyses (listwise exclusion). Two-tailed P values less than 0.05 were considered as significant. Hosmer-Lemeshow’s test was used to evaluate the goodness of fit for a multiple logistic regression model.^30^ All the analyses were run with SPSS^®^ version 10.0J.^31^

## RESULTS

Table 1 presents the prevalence of PLMS and sleep-related complaints after age adjustments. In comparison with those of the difficulty in initiation sleep or the difficulty in maintaining sleep as shown in Table 1, the prevalence of PLMS was much lower. For those who had PLMS for at least once per week in the past month, the prevalence was 7.1% (95% CI; 6.0-8.3%).

**Table 1.** Age-adjusted prevalence of periodic leg movements during sleep and sleep-related complaints in 884 Japanese adults

per week in the past month	Periodic leg movements during sleep		Difficulty in initiating sleep		Difficulty in maintaining sleep		Excessive daytime sleepiness	
	n	% (95% CI)	n	% (95% CI)	n	% (95% CI)	n	% (95% CI)
None	656	74.9 (82.9-76.9)	496	57.6 (55.4-59.8)	419	49.9 (47.7-52.2)	696	75.3 (73.3-77.2)

<1 time	150	17.9 (16.2-19.7)	177	19.8 (18.0-21.6)	139	14.7 (13.1-16.3)	105	14.1 (12.5-15.7)

1 to 2 times	56	5.8 (4.7-6.8)	115	11.7 (10.2-13.1)	159	17.4 (15.7-19.1)	63	7.8 (6.6-9.0)

3+ times	11	1.3 (0.8-1.9)	82	9.6 (8.3-11.0)	150	16.6 (14.9-18.3)	19	2.7 (2.0-3.5)

CI: confidence interval

Associated factors with PLMS were explored in the main contexts of age and sex, sleep-related complaints and medication use to aid sleep, physical and mental health status, and psychoactive substances affecting sleep. In univariate analyses, significant associations of PLMS were identified with the difficulty in initiating sleep, the difficulty in maintaining sleep, the excessive daytime sleepness, and snoring, physical health status (e.g., asthma and peptic ulcer), and mental health status (e.g., depressed mood, being under strain, difficulty concentrating, facing up to problems or overcoming difficulties, losing confidence and thinking oneself worthless). As shown in Table 2, the following factors still remained significant in a multivariate analysis; those with PLMS were more likely to experience difficulties in initiating sleep, snore during sleep, be depressed, and suffer from peptic ulcer (goodness-of-fit: chi-squared 2.404, df 4, p=0.662). Age, sex, difficulty in maintaining sleep, excessive daytime sleepness, medication use to aid sleep, and any psychoactive substance were not significantly correlated to PLMS in the multivariate model.

**Table 2.** Associated factors of periodic leg movements during sleep in 884 Japanese adults

Factor	Univariate		Multivariate	
	n	Odds ratio	n	Odds ratio
		(95% CI)		(95% CI)
Difficulty in initiating sleep				
absent	491	1.00	460	1.00
present	370	2.00 (1.46-2.74)	344	1.57 (1.11-2.20)

Difficulty in maintaining sleep				NS
absent	416	1.00		
present	443	1.69 (1.23-2.31)		

Excessive daytime sleepiness				NS
absent	687	1.00		
present	185	1.58 (1.11-2.56)		

Medication use to aid sleep				NS
no	800	1.00		
yes	72	1.47 (0.87-2.47)		

Snore				
absent	740	1.00	701	1.00
present	117	2.21 (1..47-3.32)	103	2.25 (1.43-3.53)

Depressed mood				
absent	617	1.00	569	1.00
present	252	2.25 (1.63-3.11)	235	2.18 (1.54-3.10)

Peptic ulcer				
absent	838	1.00	774	1.00
present	34	2.84 (1.42-5.66)	30	2.92 (1.35-6.32)

NOTE: All variables were entered and analyzed using the forward selection stepwise procedure (P≦0.05 as inclusion and P≧0.10 as exclusion) in a multivariate model: age, sex, marital status, education, job status, sleep-related complaints, medication use to aid sleep, tobacco, alcohol, caffeine, snore, hypertension, heart disease, diabetes mellitus, asthma, peptic ulcer, and each item of the 12-item General Health Questionnaire excluding insomnia. CI: confidence interval; NS: not significant

## DISCUSSION

The present study was the nationwide epidemiologic study to explore the prevalence and associated factors of PLMS for Japanese adults in a community setting. Among the subjects with bed partners or bedroom mates, the prevalences of PLMS were 5.8% (95% CI; 4.7-6.8%) and 1.3% (95% CI; 0.8-1.9%) for 1 to 2-times, and 3-times or greater per week in the previous month, respectively. Examining the associated factors of PLMS, the multivariate analysis revealed that difficulty in initiating sleep, snoring, depressed mood, and peptic ulcer were significantly associated with PLMS.

We found only a few community-based epidemiologic studies on PLMS or periodic limb movement disorder for comparison with the findings in the present study. According to a brief report by Kageyama et al.,^13^ the prevalence was 2-5% for females and 8-12% for males among 4,612 community residents (3,600 females and 1,012 males) in the geographically limited areas of Japan. A case definition of PLMS used in their study was based on a self-report using a self-administered questionnaire; Have you ever been told you jerk your legs or kick during sleep? (Yes/No/Sometimes). The answer of yes to the question was considered as representing PLMS. Male preponderance of PLMS was identified for the age group of 30-69 years. No age effect on prevalence of PLMS was found.

Ohayon and Roth^14^ conducted a telephone interview survey for a representative sample of 18,980 subjects aged 15 years and above in the general population of five European countries. They asked the following question; according to you or your bed partner, do you move your limbs a lot during your sleep? The prevalences were 5.9%, 5.5% and 7.8% for several times per month, one night per week, and 2 nights or more per week, respectively. They also reported 3.9% as a prevalence of periodic limb movement disorder using the minimal criteria according to the International Classification of Sleep Disorders.^7^

Ancoli-Israel et al.^12^ conducted telephone interviews to 1,865 elderly community residents in San Diego, USA. The subjects were asked whether their legs often twitch or kick during the night while they are asleep. The prevalence was 16% vs. 10% for those with and without sleep recordings. Of 420 elderly volunteers willing to have their sleep recorded, 45% showed that a myoclonus index was 5 or higher.

The prevalence across the above studies ranged 2-16%.^12^^-^^14^ Although these findings derived from different study subjects and case ascertainments could not be strictly compared, it was implied that the prevalence of PLMS in Japanese community-dwelling adults was not so high as those reported from Western countries.

Associated factors of PLMS or periodic limb movement disorder examined were not concordant among studies. For age, the recent epidemiologic community-based studies for the general population with a wide age range^13^^,^^14^ including the present study did not suggest that PLMS or periodic limb movement disorder was more common in the elderly.^11^^,^^12^ Regarding sex, the present study did not show a significant relation between PLMS and sex, but some studies reported a male dominance of PLMS or periodic limb movement disorder.^11^^-^^14^ The inconsistent findings on age and sex across studies may be partly due to the different methods used in each study (e.g., study subjects, data collection and case definitions). Further well-controlled studies are needed to clarify how aging plays a role in a mechanism of causing or developing PLMS and whether or not there is sex difference in PLMS.

The correlation between PLMS and sleep disturbances is of interest regarding the symptomatology of PLMS. Recently, Montplaisir et al.^17^ compared the frequencies of PLMS among 100 middle-aged subjects with various sleep-related disorders, and found no significant difference in PLMS among insomniacs, hypersomniacs, and healthy controls, suggesting that PLMS may not contribute to the complaints of insomnia and hypersomnia. Rather, they suggested much higher frequencies of PLMS among narcoleptic and restless leg syndrome patients. Youngstedt et al.^15^ reported no association of myoclonus index with subjective and objective sleep measures in 22 elderly volunteers with complaints of poor sleep or depression. Mendelson^16^ examined 67 periodic limb movement disorder patients and reported no significant correlation between the periodic leg movement arousal index and the measures of sleepiness. To our knowledge, there was no epidemiologic study investigating the associations of PLMS with sleep disturbances other than the present study. The present findings did not show that PLMS was significantly associated either with the difficulty in maintaining sleep or with the excessive daytime sleeping. In addition, there was no significant difference in medication use to help sleep between those with and without PLMS. Considering these findings with together, PLMS may not disrupt sleep too severely to cause nocturnal and early morning awakenings and daytime somnolence in the subjects of our study. A relation between PLMS and the difficulty in initiating sleep found in the present study may be caused by a possible coexistence of restless leg syndrome, which is often accompanied by PLMS and disturbs falling asleep.^17^^,^^32^

The findings obtained in the present study on associations or co-morbidity of PLMS with some physical and mental conditions are in agreement with those reported from other studies, such as snoring or obstructive sleep apneas^17^^-^^19^^,^^21^^,^^22^^,^^33^^,^^34^ and depressed mood.^15^^,^^21^^,^^22^ In the current study, peptic ulcer appeared as a significant correlate with PLMS, which was not reported in any pervious studies. It is too early, however, to confirm this finding because the number of those with peptic ulcers was small. Further investigation is needed.

Some limitations should be taken into account in interpreting the findings of the present study. Firstly, as noted earlier, we did not have objective data on PLMS measured with polysomnograpies or electromyographies. A validation study about sensitivity and specificity using the assessments of observers (e.g., bed partners and bed roommates) and electrophysiological tests (e.g., polysomnographies and electromyographies) is considered to implement in the near future. Secondly, the subjects used for this study were targeted only to those who had bed partners or bedroom mates. We checked statistical significance in sociodemographic characteristics between the subjects with and without bed partners or bedroom mates. There was no sex difference, but the former were less likely to be younger or older, and more likely to be married, employed, and finish high school, college or university than the latter. Thus, there is a possibility of the present findings distorted due to such selection bias. Thirdly, the goodness-of-fit of the model was not satisfactory, because the data in each stratum of the model was so sparse that the model might not become stable. The larger number of subjects is necessary for the future study. Lastly, our cross-sectional study design could not refer to a cause-effect relation between PLMS and its risk factors.

In summary, the present study provides us with the preliminary evidence on prevalence and associated factors of PLMS for Japanese adults with bed partners or bedroom mates in a community setting. The results in this study deserve emphasis for promoting further research of PLMS or periodic limb movement disorder with regard to its etiology and symptomatology.
